# Post-Pleistocene range expansion of the recently imperiled eastern little brown bat (*Myotis lucifugus lucifugus*) from a single southern refugium

**DOI:** 10.1002/ece3.20

**Published:** 2011-10

**Authors:** Michael D Dixon

**Affiliations:** University of Minnesota

**Keywords:** Demographic history, *Myotis lucifugus*, niche modeling, Pleistocene refugia, population growth, range expansion

## Abstract

*Myotis lucifugus*, once among the most widespread and common bats in North America, has been forecast to be extirpated east of the Rockies in as few as 16 years by the spread of white-nose syndrome. Recent genetic research has demonstrated that this species is paraphyletic and part of a broader species complex; however, only one lineage (*Myotis lucifugus lucifugus [M. l. lucifugus]*) is present in eastern North America. I used molecular tools and niche modeling to validate this and investigate the role that historical biogeography has played in the phylogenetic and population genetic structure of this species to determine if the eastern subspecies represents an evolutionarily distinct population.

To establish the genetic structure within *M. l. lucifugus*, I densely sampled maternity colonies in Minnesota and sequenced 182 individuals for a portion of cytochrome b. Phylogenetic reconstruction and a haplotype network were used to infer the relationships among mitochondrial haplotypes. Population growth statistics were calculated to determine if there was evidence of significant expansion, and an environmental niche model (ENM) was constructed based on conditions during the last glacial maximum (LGM) to illustrate potential glacial refugia. All individuals derived from a single mitochondrial lineage. Genetic evidence points to population growth starting approximately 18 kya. ENM results show that there was likely a single large southern refugium extending across the southeastern United States and possibly several isolated refugia in western North America. *Myotis lucifugus lucifugus* likely maintained both a large range and a large population during the peaks of the glacial cycles, and its population appears to have expanded following the retreat of the Laurentide ice sheet. This imperiled lineage likely diverged in isolation from other members of the *M. lucifugus*/western long-eared *Myotis* during the Pleistocene.

## Introduction

The Wisconsin glaciation, as well as earlier glacial periods, caused substantial expansions, contractions, and shifts in the ranges of plants and animals of North America (Pielou 1991) and throughout the temperate regions. This caused strong but sometimes latent effects on the evolutionary trajectories of these species. This has resulted in patterns of lineage endemism and speciation among glacial refugia, though the pattern is often quite complex (see review in Soltis et al. 2006). Phylogeographic studies of mammal distributions in the Pleistocene have alternately found genetic structure pointing to refugia in unexpected places (Deffontaine et al. 2005) and a surprising lack of genetic structure in other species (Moncrief et al. 2010). An understanding of how past distributions influenced the present diversity within and among lineages is important for understanding both the processes that promote the generation of biodiversity and the range of genetic variation within species, which is particularly important in taxa of conservation concern.

**Figure 1:**
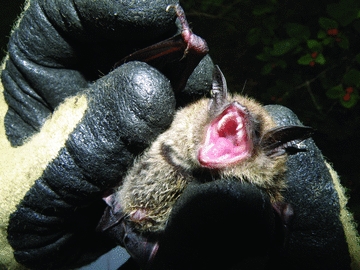
A female little brown bat *M. lucifugus* captured at a maternity roost in Voyageurs National Park, MN, USA. Photo by Bill Severud, NPS.

Research to date has not explored the potential distribution of *Myotis lucifugus* (Fig. 1) during the last glacial maximum (LGM). *Myotis lucifugus* is among the most widely distributed bats in North America (Reid 2006) and one that has been quite well studied (Fenton and Barclay 1980), with a range extending from the coastal southeastern United States through the Yukon and central Alaska. As many as six subspecies are recognized (Fenton and Barclay 1980), though *M. l. occultus* appears to represent an independent lineage (Piaggio et al. 2002) and neither nuclear genes nor morphology appear to be able to diagnose *M. l. carissima* and *M. l. lucifugus* (Lausen et al. 2008). Recent genetic research indicates that “*Myotis lucifugus*” as it has historically been defined may be paraphyletic, with members of the western long-eared bats sister to some recognized subspecies of *M. lucifugus* (Dewey 2006; Carstens and Dewey 2010). The relationships among these lineages are still poorly understood and require further research, but it has been accepted that a single, monophyletic mitochondrial lineage of the little brown bat, *M. l. lucifugus*, ranges across North America east of the Great Plains (Dewey 2006). This lineage is currently being annihilated by white-nose syndrome, a disease which models predict will cause the species to be regionally extinct in eastern North America in as few as 16 years (Frick et al. 2010). This has resulted in a group of prominent bat biologists and conservation organizations petitioning the U.S. Fish and Wildlife Service for an emergency listing as an endangered species under the federal Endangered Species Act (Kunz and Reichard 2010), and states have begun undertaking similar action (Munkwitz et al. 2010). There are many cases in which thorough examinations of “subspecies” have revealed that there was little genetic basis for that designation (Zink 2004) or conversely that there were actually more species than previously recognized that were in need of protection due to undescribed diversity (Rojas-Soto et al. 2010). Therefore, research that can help elaborate the boundaries of this species is increasingly prudent as we move to determine what taxon or taxa are in need of protection, and to ensure that conservation efforts and resources are expended judiciously.

Here, I present the results of a study that explores how glacial refugia during the Pleistocene may have influenced the phylogenetic and population genetic structure in this specie(s), with particular emphasis on the purportedly phylogenetic eastern subspecies *M. l. lucifugus*.

## Methods

### Sample collection

As part of a broader study of the population genetic structure in this species, female *M. lucifugus* were sampled at 12 maternity colonies were throughout the state of Minnesota during the summers of 2010 and 2011. These colonies ranged from 5 to 585 km apart. Three of the sampled colonies were in or adjacent to Voyageurs National Park (MI: 48°37″17′N, 93°12″33′W; RLC: 48°35″88′N, 93°09″04′W; KF: 48°30″05′N, 92°38″64′W), six separate colonies were sampled in Gooseberry Falls (GF: 47°08″57′N, 91°28″28′W), Itasca (IT: 47°13″04′N, 95°11″31′W), St. Croix (SC: 45°56″65′N, 92°36″24′W), William O'Brien (WO: 45°13″06′N, 92°45″51′W), Whitewater (WW: 44°03″74′N, 92°02″71′W), and Great River Bluffs (GRB: 43°56″25′N, 91°24″31′W) State Parks, one colony was in Doyle-Kennefick Regional Park (DK: 44°36″49′N, 93°26″15′W), and two colonies were in private residences (NF: 44°29″61′N, 93°6″60′W; LV: 44°42″84′N, 93°18″51′W). Bats were captured in mist nets during emergence from roost structures or hand collected from bat houses or from buildings during the day. Colonies were only sampled once to avoid the possibility of sampling the same individual twice. A sanitized holding bucket was used to contain bats for up to 1 hour to facilitate processing. Individuals were sexed and weighed, their reproductive status recorded, and they were aged by viewing the wings with a backlight to determine the presence of visible cartilaginous areas of the finger bones (Brunet-Rossini and Wilkinson 2009). Only adult females were included in the analyses described below.

A sterile 3-mm biopsy punch was used to take a tissue sample from the plagiopatagium of each bat. Wing punches were performed on a gridded, sterilized board to minimize risk of cross-infection of bats or cross-contamination of samples, and a new biopsy punch was used for each bat. Wing punch wounds in lactating female *M. lucifugus* heal within approximately 2 weeks (Weaver et al. 2009), so the use of this technique to gain genetic data is likely fairly benign. Samples were placed immediately into 100% ethanol and then stored at –80 °C pending lab work. It has been suggested to take a voucher specimen when sampling with wing punches in case of problems arising from field misidentification (Simmons and Voss 2009), however, I chose not to take voucher specimens because of the relatively small size of the sampled maternity colonies (12–200 individuals) and as only one lineage of the *M. lucifugus*/*evotis* species complex is thought to occur in eastern North America (Dewey 2006; Carstens and Dewey 2010). Individuals were released following sampling and were handled in accordance with guidelines of the American Society of Mammalogists (Gannon and Sikes 2007) and the Institutional Animal Care & Use Committee of the University of Minnesota.

### DNA extraction, amplification, and sequencing

DNA was extracted from wing punches using a Qiagen DNEasy Blood & Tissue Kit (Qiagen Inc., Valencia, CA, USA) following the manufacturer's protocol, except that tissue samples were digested for up to 48 h in lysis buffer with 20–40 µL of proteinase K. A 654 base-pair (bp) fragment of the mitochondrial gene cytochrome b was amplified and sequenced for each individual using the forward primer F504 (GTGAATCTGAGGTGGCTTTTCCG) and the reverse primer R1181 (CATCTCCGGTTTACAAGACCAGT). Polymerase chain reaction (PCR) was carried out in 12.5-µL reaction volumes using 1 µL of undiluted template DNA, 0.5 µL of each primer, 6.25-µL GoTaq Master Mix (Promega Inc., Madison, WI, USA), and 4.25 µL of nuclease-free water. PCR began with an initial denaturing step of 2 min at 95°C, followed by five cycles each of 30 sec at 56°C, 55°C, and 54°C, then 25 cycles of 30 sec at 53°C, and concluded with a final elongation step of 7 min at 72°C. PCR products were cleaned of unincorporated nucleotides prior to sequencing using Exonuclease I and Shrimp Alkaline Phosphatase (Hanke and Wink 1995). PCR products were sequenced in both directions with amplification primers using an ABI 3730 automated sequencer (Applied Biosystems Inc., Carlsbad, CA, USA). Sequencher 4.7 (GeneCodes Inc., Ann Arbor, MI) was used to compile and edit the sequences. All sequences have been deposited in GenBank (JF899346–JF899527).

### Genetic data analysis

I reconstructed a phylogeny for my samples to confirm that they comprised a single mitochondrial lineage. The presence of multiple mitochondrial lineages in the sample would suggest that the subspecies was isolated in multiple refugia in the past, while a single lineage would support those individuals having arisen in a single refugium. I used the program TCS 2.1 to create a minimum spanning network to reconstruct the hypothetical evolutionary relationships of mitochondrial haplotypes. To understand the relationships among the clades detected in the haplotype network, as well as to confirm that all of my samples were from *M. lucifugus* rather than the grossly similar *M. setentrionalis*, I constructed a maximum likelihood tree for the haplotypes in Garli 0.951 using a Genbank cytochrome b sequence of *M. septentrionalis* (AY883911) as an outgroup. I used MrModeltest (Nylander 2004) to identify the best fitting model of nucleotide substitution, HKY +Г. Support for nodes between branches was calculated using 1000 bootstrap replicates.

To test for population growth, I used Arlequin 3.11 (Excoffier et al. 2005) to generate mismatch distributions that compare the observed number of pairwise differences among individuals compared to the expected numbers of pairwise differences if the population size had historically been constant or if it had increased or decreased (Rogers and Harpending 1992). I also calculated Fu's *F_s_* (Fu 1997) and Tajima's *D* (Tajima 1989), negative values of which can indicate population expansion. Because these negative values can also indicate selection, I calculated F* and D* (Fu and Li 1993) using DnaSP 5.10 (Rozas et al. 2009). If these values are not statistically significant but Fu's *F_s_* is, the data support population expansion, while the opposite pattern would be evidence of selection (Fu 1997).

To complement these population growth tests, and to test whether that growth followed the close of the Wisconsin glaciations, I used BEAST v1.6.1 to produce a Bayesian skyline plot (Drummond et al. 2005), which uses a coalescent method to estimate changes in the effective population size over time given genetic data and user-specified parameters to characterize sequence evolution. A likelihood-ratio test failed to reject a molecular clock hypothesis for these data so a strict clock was used, scaled with the *Myotis*-calibrated divergence rate of 4.8% per million years (Ruedi and Mayer 2001). Operators were left at default values and 10 × 10^6^ Markov Chain Monte Carlo steps were used.

If the measures of population growth were insignificant and/or the Bayesian skyline plot were indicated a more recent or more ancient period of population change, it would indicate that the population had not undergone a period of expansion following the close of the Pleistocene.

### Environmental niche modeling

To explore how past environmental change and resulting isolation in glacial refugia may have influenced the population genetic structure of this species, I used the correlative environmental niche model (ENM) technique implemented in Maxent (Phillips et al. 2006). Maxent models the probability that a given pixel is suitable for a species given the environmental conditions at that pixel as well as those across pixels where the species is known to occur. This probability is expressed as a map depicting the species’ predicted range. These environmental conditions can be any categorical or continuous variable that the user chooses, but because my purpose was to hindcast the distribution of *M. lucifugus* during the LGM, I was limited to those variables for which data are available for that time period. I chose to use the 2.5 arc-minute resolution BIOCLIM variables for the present and LGM (the latter data were based on the Community Climate System Model) from the Worldclim database (Hijmans et al. 2005) because these climatic variables (e.g., temperature seasonality) have been found to be related to the ecological and physiological tolerances of organisms, and thus effective at predicting the past and present ranges of species using Maxent (Hijmans and Graham 2006). In addition, niche models based on reconstructed paleoclimate data consistently produce projected LGM distributions that are concordant with other data sources (Waltari et al. 2007). Maxent implicitly performs model selection and choosing among variables in advance using a model selection procedure such as stepwise regression can degrade the performance of the model (Elith et al. 2010), so I used the full set of 19 bioclimatic variables.

Locality information for presence training data were compiled from the MaNIS database of museum specimens (manisnet.org), correspondence with additional museums whose collections are not georeferenced, and communication with researchers who had captured and positively identified *M. lucifugus* during fieldwork in portions of Canada that are poorly represented in museum collections. I removed localities that occurred in what was likely to be the range of *M. occultus* rather than *M. lucifugus*. Because sampling bias and intensity can strongly influence model output and lead to overfitting, I selected only localities that were greater than 10 km apart (R. Anderson, pers. comm. 2010). A total of 360 localities was included, of which 10% were randomly reserved to serve as test data for the model. The model was validated using the area under the curve (AUC) for training and test data. AUC refers to the Receiver Operating Curve, a commonly used metric of distribution model evaluation that essentially measures how successfully a model discriminates between sites where a species is present and where it is absent (Elith et al. 2006).

## Results

### Genetic data

**Figure 2:**
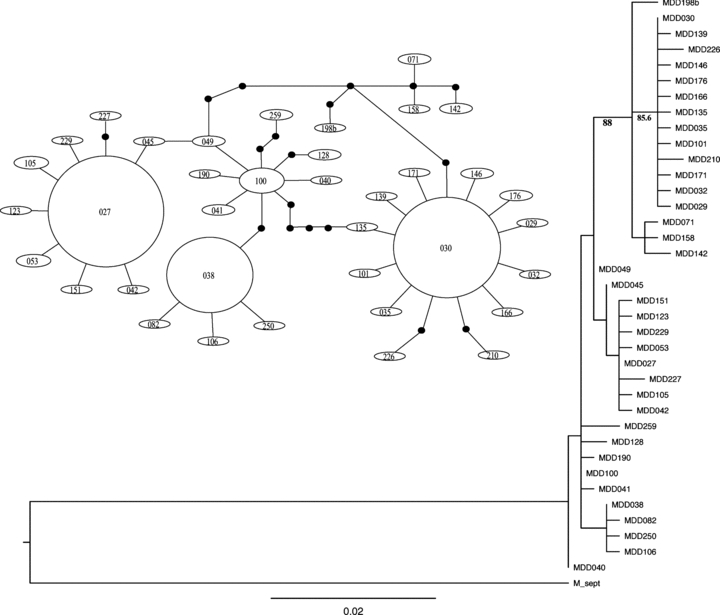
Haplotype network and maximum likelihood tree based upon 654-base fragment of cytochrome b. In the haplotype network, the size of elipses indicates the relative number of individuals present in the population who carry that haplotype. Black nodes represent intermediate mutations that are not represented in the sample population. Note that numbers simply correspond to a representative sample and are not indicative of any geographic relationship among haplotypes. Bootstrap values above 80 are presented in bold at the supported nodes in the likelihood tree.

A total of 37 mitochondrial haplotypes was recovered from the 182 bats. The vast majority of these individuals had one of four haplotypes, so most of the genetic diversity comprised one, two, or three individuals that possessed a sequence differing by one or two mutations. This resulted in a series of connected star-like patterns in the haplotype network (Fig. 2), representing a handful of closely related lineages. This star-like pattern is suggestive of rapid population expansion (Joy et al. 2003), as it shows that a large number of single-point mutations are being maintained in the population rather than lost due to drift as would be expected when neutral mutations enter a population at low frequency in a stable population. The relationship among these haplotypes is also apparent from the maximum-likelihood tree (Fig. 2). Poorly supported nodes were left in place for the purposes of comparing the concordance between the tree and the network, but if one were to collapse these nodes there would be a single clade nested within a polytomy, suggesting that the samples are all derived from a single mitochondrial lineage, or at least illustrating the absence of a second molecularly diagnosable lineage in the sampled population.

**Figure 3:**
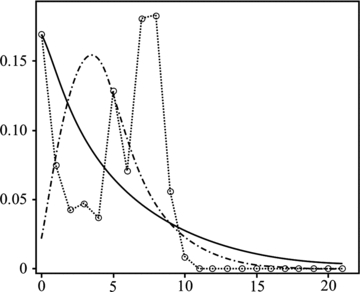
Mismatch distribution based upon cytochrome b. Horizontal axis is the number of nucleotide differences in pairwise comparisons among samples; vertical axis is the proportion of pairwise comparisons with that number of differences. The solid line indicates the expected distribution if the population had historically maintained constant size, the dash-dot line indicates the expectation if the population had expanded, and the short dashed line is the observed pattern among *M. lucifugus* in Minnesota.

**Figure 4:**
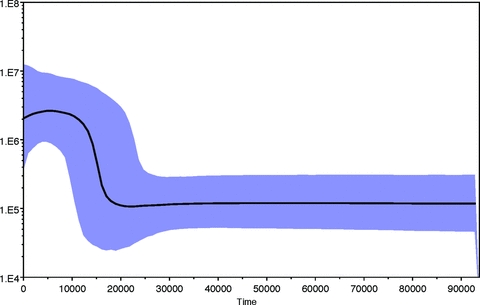
Bayesian skyline plot of the median effective population size of *M. lucifugus* (log N_e_) versus absolute time in years before present, scaled by the *Myotis*-specific mutation rate determined by Castella et al. 2000. Shaded area indicates 95% confidence intervals surrounding the median.

The mismatch distribution (Fig. 3) exhibited a bimodal distribution that was very different from that expected under a constant population size, and its low and (marginally) insignificant raggedness index (*r*= 0.0526, *P*= 0.06) fails to reject the null hypothesis of population growth, if just barely. Tajima's *D* was negative, though insignificantly so (–1.16532, *P*= 0.10700), and Fu's *F_s_* was significantly negative (–12.88349, *P*= 0.00400). Neither Fu and Li's D* (–0.06838, *P*= 0.46800) nor F* (–0.11037, *P*= 0.51900) were significant, so these neutrality tests likely indicate demographic expansion in the sampled population rather than natural selection. The Bayesian skyline plot (Fig. 4) strongly suggests rapid population expansion beginning approximately 18 kya.

### Pleistocene niche model

**Figure 5:**
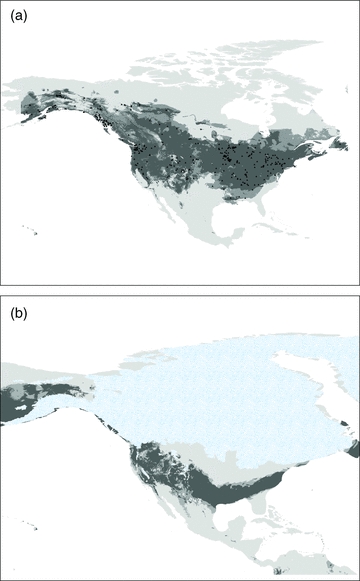
Environmental niche models of: (A) The present distribution of *M. lucifugus* based on 360 known occurrence records; and (B) A projection of suitable habitat for the species during the last glacial maximum (18 kya). Presence localities are shown as dark triangles. The darkness of shading indicates the relative suitability: light gray is unsuitable, intermediate represents the minimum training presence threshold, and dark gray represents the more conservative fixed cumulative value 10 threshold. The stippled area in (B) represents the ice extent at the LGM (data from Dyke, 2004).

The current distribution predicted by Maxent (Fig. 5A) very closely mirrors the accepted range of *M. lucifugus* (Reid 2006), though it seems to under-predict the presence of the species in northern Québec and Newfoundland, most likely because of sampling bias. The AUC for training data was 0.903 and for test data was 0.872, both much higher than the random prediction of 0.5. The variables with the most important effect on AUC following permutation of their values in training and background data were the minimum temperature of the coldest period (25.3%), precipitation of the driest period (11.8%), mean temperature of the driest quarter (10.5%), and the mean temperature of the coldest quarter (9.3%). Permutation of each of the remaining variables changed the overall AUC by 5% or less. However, because many of the climatic variables are correlated, interpretation of the effects of permuting individual variables should proceed cautiously.

The projection for the species range during the LGM (Fig. 5B) shows a large contiguous band of suitable habitat across the southeastern United States and lower Great Plains, with fragmented or tenuously connected habitat scattered throughout western North America. The presence of suitable habitat in Newfoundland is questionable, as it was at least partially glaciated during the LGM, but the extent of its ice cover is debated (Pielou 1991) and it was not included in the dataset of glaciated area used in this figure (Dyke 2004). Intriguingly, the model also shows strong support for a widespread refugium in unglaciated Beringia.

## Discussion

The physical (and thus likely the ecological) conditions of North America have never been completely homologous with current conditions, nor does the current interglacial represent a return to “things as they were” (Pielou 1991). Thus, it is appropriate to critically view the projection of species’ ranges into past or future climates in which there may be combinations of climate conditions without a contemporary analog. That said, the niche model does appear to accurately approximate the range of the species in the present day, including its absence in portions of the Great Plains, California's Central Valley, and the southeastern United States; these bioclimatic variables are likely to have some bearing on the species physiological tolerances during the LGM. Genetic data and the predictions from LGM ENM appear to be strongly concordant. The lack of deep genetic divisions within the eastern mitochondrial lineage/subspecies *M. l. lucifugus* from previous, comprehensive phylogenetic analyses and this denser sample of limited area are consistent with the model output, which predicts a large continuous refuge in the lower Great Plains and the “Floridian” thermal enclave reviewed in Russell et al. (2009). Numerous accounts of fossil evidence support the presence of several species of *Myotis* including *M. lucifugus,* and members from many other genera in this region during Pleistocene glaciations (Choate and Hall 1967; Martin 1972; Czaplewski 1993; Russell et al. 2009), and thus it probably represents an important biogeographic area for the maintenance of diversity in multiple bat taxa. It is likely that this refugium maintained a substantial population for the species, as the tests for population expansion in this study gave mixed results, with more sensitive Fu's *F_s_* and Bayesian skyline plot indicating population expansion, but only marginal support for it based upon Tajima's *D* and the mismatch distribution. This large refugium is supported by findings for the southern red-backed vole, which was found to have had a large, stable population in its southern-central U.S. glacial refugium in the same location as that predicted in this study (Runck and Cook 2005).

Unsurprisingly, it appears that *M. lucifugus* likely persisted in several isolated refugia in the intermountain and coastal western North America. These are largely concordant with those refugia suggested by phylogeographic research in other species, including western North American shrews (Demboski and Cook 2001), voles in southeast Alaska and coastal Canada (Conroy and Cook 2000), and black bears (Byun et al. 1997) and weasels (Byun et al. 1999) in the vicinity of the Queen Charlotte Islands. The suitable habitat predicted in Beringia is interesting, in that it is thought that Beringia was mostly either a steppe habitat (Zazula et al. 2003) or tundra during that period (Pielou 1991) and *M. lucifugus* is generally associated with proximity to some type of tree cover. However, it is possible that small spruce woodlands persisted there during the height of the glacial episodes (Heuser 1983; Zazula et al. 2009), and thus it is not impossible that Beringia harbored both a suitable climate and trees which could have served as roosts. However, discovering the role of isolated Pacific coastal and Beringian refugia in producing the current array of lineages/species within the *M. lucifugus*/western long-eared bat complex will require a more thorough phylogeographic study, such as that undertaken to test refugial hypotheses in singing voles (Weksler et al. 2010). The western subspecies of *M. lucifugus* (*alascensis, carissima, relictus*) and the apparently closely related *M. evotis*, *M. keenii*, and *M. thysanodes* form a paraphyletic group that occurs both in sympatry and allopatry throughout western North America, and most of the divergence within this clade appears to have occurred within the Pleistocene (Carstens and Dewey 2010). Given the probable fragmentation among Pleistocene glacial refugia that was predicted in this study, it is likely that much of the genetic differentiation observed by Carstens and Dewey occurred in allopatry during repeated Pleistocene glacial advances. Previous research has shown that closely related *Myotis* bats are capable of rapidly evolving morphological adaptations that allow them to form convergent ecomorphs to exploit open niche space (Ruedi and Mayer 2001; Stadelmannn et al. 2007). Since, much of what is now the *M. lucifugus–thysanodes–evotis–keenii* species complex went through repeated vicariance events followed by range expansion, there could well have been the evolution of short- and long-eared *M. lucifugus* ecomorphs and lineages that have never fully speciated, either because of recurring gene flow, incomplete lineage sorting, or some combination thereof. Disentangling these phenomena is notoriously challenging. Different methods of estimating gene flow have given quite different results in this species complex (Carstens and Dewey 2010). However, there is evidence of contemporary nuclear gene flow in the hybrid zone between the fairly well-resolved mitochondrial lineages of *M. l. lucifugus* and *M. l. carissima* (Lausen et al. 2008), and thus it is possible, even probable, that the boundaries between these recently divergent lineages are fuzzier than current taxonomy would suggest. The presence of eastern mtDNA haplotypes in western North America (Dewey 2006) most likely derives from either incomplete lineage sorting or modern or past interglacial dispersal as there appears to have been less contact between areas of suitable habitat in the glacial periods than during the present era.

The finding of weak but significant population expansion in *M. l. lucifugus* approximately coinciding with the end of the Wisconsin glaciation was expected, as many taxa dramatically expanded their ranges (Pielou 1991) and thus their populations as the Laurentide ice sheet retreated. The pace of this expansion is unclear, though there is fossil evidence of *M. lucifugus* in Pennsylvania just over 11 kya (Martin 1972). The rapidity and recentness of post-Pleistocene expansion has left conflicting patterns of phylogeographic structure in eastern North America within other mammal species that expanded from southeastern forest refugia. The eastern fox squirrel (Moncrief et al. 2010) shows no geographic mitochondrial structure across its range, while the northern and southern flying squirrels display a more classic pattern of increasing genetic diversity nearer to the likely glacial refugiua (Arbogast et al. 2005; Arbogast 2007). Whether or not a detailed phylogeographic study of *M. lucifugus* would uncover the latter or former pattern, or something different altogether, is outside the scope of this study. However, it is worth noting that the sampled populations in Minnesota are in the middle of the continental range in an area that was nearly entirely glaciated during the LGM, and yet mitochondrial haplotype and nuclear microsatellite diversity in each sampling locality was quite high and there is no isolation by distance detectable among the sites that are as far as 600 km apart (Dixon, in press) probably due to the species’ ability to disperse over hundreds of kilometers (Humphrey and Cope 1976), suggestive of the maintenance of high genetic diversity in its glacial refugium, rapid expansion, and possibly frequent modern dispersal.

These results suggest that the little brown bat *M. lucifugus* maintained a relatively large geographic distribution during the Pleistocene glaciations, and that habitat expansions and contractions from glacial refugia in western North America likely explain the complex evolutionary history of the *M. lucifugus*/western long-eared bat species complex. The eastern subspecies *M. l. lucifugus* likely persisted in a single refugium in which it has evolved independently of other lineages. The facts that both sparse but widespread sampling in previous studies (Dewey 2006; Carstens and Dewey 2010) and dense, local sampling in this study found the eastern subspecies to be monophyletic, and that it appears to have diverged from the other lineages in a large southeastern refugium, argue that this widespread subspecies may be an evolutionarily significant unit of *M. lucifugus*. If indeed the U.S. Fish and Wildlife Service deems in necessary to list *M. lucifugus* under the U.S. Endangered Species Act, then that analysis should consider managing *M. l. lucifugus* as a distinct population segment. While one could certainly debate the potential benefits of Endangered Species Act protection in this instance, I think that there is no question that the potential extinction of this formerly abundant and widespread subspecies would represent a tragic loss for North American biodiversity.
